# Supplementary material for: Phylogenomic analyses reveal a Gondwanan origin and repeated out of India colonizations into Asia by tarantulas (Araneae: Theraphosidae)
Source: PeerJ. 2021 Apr 6;9:e11162. doi: 10.7717/peerj.11162 (PMC8034372; doi:10.7717/peerj.11162)
Supplement: Supplemental Information 5 — Country-level data was accessed from the World Spider Catalog (reference per manuscript body). [file peerj-09-11162-s005.docx]

| **SPECIES** | **SAMPLE ID** | **SRA RUN** | **GEOGRAPHIC RANGES** |
| --- | --- | --- | --- |
| *Acanthoscurria geniculata* | SAMN02378633 | SRR1024075 | **D** (Brazil) |
| *Aliatypus coylei* | [SAMN02837049](https://www.ncbi.nlm.nih.gov/biosample/SAMN02837049) | SRR1514876 |  |
| *Antrodiaetus unicolor* | [SAMN02837056](https://www.ncbi.nlm.nih.gov/biosample/SAMN02837056) | SRR1514897 |  |
| *Aphonopelma johnnycashi* | SAMN02836947 | SRR1514871 | **D** (USA) |
| *Aptostichus atomarius* | [SAMN02837057](https://www.ncbi.nlm.nih.gov/biosample/SAMN02837057) | SRR1514885 |  |
| *Aptostichus stephencolberti* | [SAMN02837047](https://www.ncbi.nlm.nih.gov/biosample/SAMN02837047) | SRR1514874 |  |
| *Brachythele longitarsus* | [SAMN02837048](https://www.ncbi.nlm.nih.gov/biosample/SAMN02837048) | SRR1514875 |  |
| *Caribena laeta* | SAMN11475840 | SRR8944278 | **D** (Puerto Rico, Cuba, US Virgin Islands) |
| *Caribena versicolor* | SAMN11475841 | SRR8944282 | **D** (Martinique) |
| *Catumiri* sp. | SAMN11475842 | SRR8944288 | **D** (Argentina) |
| *Cyclocosmia truncata* | [SAMN02837045](https://www.ncbi.nlm.nih.gov/biosample/SAMN02837045) | SRR1514884 |  |
| *Cyriopagopus lividus* | SAMN11475847 | SRR8944277 | **B** (Myanmar, Thailand) |
| *Damarchus* sp. | SAMN04453350 | SRR3144092 |  |
| *Ephebopus cyanognathus* | SAMN11475845 | SRR8944275 | **D** (French Guiana) |
| *Eriophora transmarina* | [SAMN08668850](https://www.ncbi.nlm.nih.gov/biosample/SAMN08668850) | SRR6967881 |  |
| *Haplocosmia* sp. | SAMN11475846 | SRR8944280 | **A, B** (Nepal / Himalayas) |
| *Hebestatis theveneti* | SAMN02837036 | SRR1514887 |  |
| *Heteroscodra maculata* | SAMN11475848 | SRR8944261 | **E** (West / Central Africa) |
| *Hysterocrates gigas* | SAMN11475849 | SRR8944262 | **E** (Cameroon) |
| *Idiops bersebaensis* | [SAMN02837059](https://www.ncbi.nlm.nih.gov/biosample/SAMN02837059) | SRR1514907 |  |
| *Lasiodora parahybana* | SAMN11475850 | SRR8944263 | **D** (Brazil) |
| *Linothele fallax* | SAMN11475851 | SRR8944264 |  |
| *Liphistius malayanus* | [SAMN02597590](https://www.ncbi.nlm.nih.gov/biosample/SAMN02597590) | SRR1145736 |  |
| *Megahexura fulva* | [SAMN02837037](https://www.ncbi.nlm.nih.gov/biosample/SAMN02837037) | SRR1514891 |  |
| *Microhexura montivaga* | [SAMN02837040](https://www.ncbi.nlm.nih.gov/biosample/SAMN02837040) | SRR1514890 |  |
| *Monocentropus balfouri* | SAMN11475852 | SRR8944273 | **E** (Socotra Island) |
| *Neoholothele incei* | SAMN11475853 | SRR8944287 | **D** (Trinidad & Tobago, Venezuela) |
| *Omothymus schioedtei* | SAMN11475843 | SRR8944271 | **B** (Malaysia) |
| *Omothymus* sp. “Hati Hati” | SAMN11475844 | SRR8944281 | **B** (Indonesia) |
| *Paratropis* sp. | [SAMN02836945](https://www.ncbi.nlm.nih.gov/biosample/SAMN02836945) | SRR1514893 |  |
| *Pelinobius muticus* | SAMN11475855 | SRR8944274 | **E** (Kenya, Tanzania) |
| *Phlogiellus inermis* | SAMN11475856 | SRR8944272 | **B, C** (Malaysia to Lombok Indonesia) |
| *Phormictopus atrichomatus* | SAMN11475857 | SRR8944270 | **D** (“probably Hispanola”) |
| *Pionothele* n. sp. | [SAMN02837051](https://www.ncbi.nlm.nih.gov/biosample/SAMN02837051) | SRR1514906 |  |
| *Poecilotheria vittata* | SAMN11475858 | SRR8944269 | **A** (India) |
| *Promyrmekiaphila clathra* | [SAMN02837043](https://www.ncbi.nlm.nih.gov/biosample/SAMN02837043) | SRR1514896 |  |
| *Psalmopoeus cambridgei* | SAMN11475860 | SRR8944279 | **D** (Trinidad) |
| *Psalmopoeus irminia* | SAMN11475861 | SRR8944267 | **D** (Venezuela, Guiana, Brazil) |
| *Pterinochilus chordatus* | SAMN11475862 | SRR8944268 | **E** (East Africa) |
| *Pterinochilus lugardi* | SAMN11475863 | SRR8944284 | **E** (Southern, East Africa) |
| *Rhianodes atratus* | SAMN15032352 | SRR11860498 |  |
| *Selenocosmia javanensis* | SAMN11475864 | SRR8944260 | **B, C** (Malaysia to Sulawesi Indonesia) |
| *Sphodros rufipes* | [SAMN02837046](https://www.ncbi.nlm.nih.gov/biosample/SAMN02837046) | SRR1514908 |  |
| *Stromatopelma calceatum* | SAMN11475865 | SRR8944276 | **E** (West Africa) |
| *Tapinauchenius violaceus* | SAMN11475867 | SRR8944283 | **D** (French Guiana, Brazil) |
| *Thrigmopoeus* sp. | SAMN11475866 | SRR8944286 | **A** (India) |
| *Trichopelma laselva* | SAMN02837052 | SRR1514881 | **D** (Costa Rica) |
