# Supplementary material for: Phylogenomic analyses reveal a Gondwanan origin and repeated out of India colonizations into Asia by tarantulas (Araneae: Theraphosidae)
Source: PeerJ. 2021 Apr 6;9:e11162. doi: 10.7717/peerj.11162 (PMC8034372; doi:10.7717/peerj.11162)
Supplement: Supplemental Information 6 [file peerj-09-11162-s006.docx]

| **SPECIES** | **READS** | **CONTIGS** | **AV. LENGTH** | **TRANS** **DECODER** |
| --- | --- | --- | --- | --- |
| *Rhianodes atratus* | 23881499 | 250639 | 538.97 | 67386 |
