# Supplementary material for: Phylogenomic analyses reveal a Gondwanan origin and repeated out of India colonizations into Asia by tarantulas (Araneae: Theraphosidae)
Source: PeerJ. 2021 Apr 6;9:e11162. doi: 10.7717/peerj.11162 (PMC8034372; doi:10.7717/peerj.11162)
Supplement: Supplemental Information 7 [file peerj-09-11162-s007.docx]

| **AREA 1** | **AREA 2** |
| --- | --- |
| Americas (needs “+ Africa” to connect to:) | India  Asia  Oceania |
| Africa (needs “+ India” to connect to:) | Asia  Oceania |
