# Supplementary material for: Phylogenomic analyses reveal a Gondwanan origin and repeated out of India colonizations into Asia by tarantulas (Araneae: Theraphosidae)
Source: PeerJ. 2021 Apr 6;9:e11162. doi: 10.7717/peerj.11162 (PMC8034372; doi:10.7717/peerj.11162)
Supplement: Supplemental Information 8 [file peerj-09-11162-s008.docx]

| **Node Number** | **Estimated Age** | **95% HPD Max** | **95% HPD Min** |
| --- | --- | --- | --- |
| 1 | 378.5 | 416.5 | 359.5 |
| 2 | 363 | 390.5 | 342.5 |
| 3 | 295.5 | 298 | 295 |
| 4 | 226 | 226.5 | 222.5 |
| 5 | 225 | 225.5 | 221 |
| 6 | 132 | 132.5 | 128.5 |
| 7 | 196.5 | 197.5 | 189.5 |
| 8 | 191.5 | 193 | 186 |
| 9 | 187 | 188 | 181.5 |
| 10 | 174 | 175 | 168.5 |
| 11 | 143.5 | 144 | 139 |
| 12 | 168 | 169 | 163 |
| 13 | 136.5 | 137 | 132 |
| 14 | 51.5 | 52 | 49.5 |
| 15 | 185 | 186 | 179.5 |
| 16 | 142.5 | 143.5 | 138 |
| 17 | 127 | 128 | 123 |
| 18 | 156.5 | 157.5 | 152 |
| 19 | 140 | 141.5 | 136 |
| 20 | 119 | 120 | 115.5 |
| 21 | 111 | 112 | 107.5 |
| 22 | 57 | 58 | 55.5 |
| 23 | 45 | 45.5 | 43.5 |
| 24 | 109 | 111 | 106.5 |
| 25 | 107 | 108 | 103.5 |
| 26 | 98 | 99 | 95 |
| 27 | 57 | 57.5 | 55 |
| 28 | 47 | 47.5 | 45.5 |
| 29 | 99 | 100 | 96 |
| 30 | 72 | 73 | 69.5 |
| 31 | 28 | 28.5 | 27 |
| 32 | 16 | 16.5 | 15 |
| 33 | 71.5 | 72 | 69 |
| 34 | 34.5 | 35 | 33 |
| 35 | 23.5 | 24 | 22 |
| 36 | 100 | 101 | 97 |
| 37 | 95 | 96 | 92.5 |
| 38 | 89 | 90 | 86.5 |
| 39 | 77 | 78 | 74.5 |
| 40 | 42 | 42.5 | 40.5 |
| 41 | 39 | 39.5 | 37.5 |
| 42 | 18.5 | 18.5 | 17.5 |
| 43 | 41.5 | 42 | 39.5 |
| 44 | 55.5 | 56 | 53.5 |
| 45 | 50.5 | 51 | 48.5 |
| 46 | 29.5 | 30 | 28.5 |
