# Supplementary figures and images for: Phylogenomic analyses reveal a Gondwanan origin and repeated out of India colonizations into Asia by tarantulas (Araneae: Theraphosidae)
Source: PeerJ. 2021 Apr 6;9:e11162. doi: 10.7717/peerj.11162 (PMC8034372; doi:10.7717/peerj.11162)

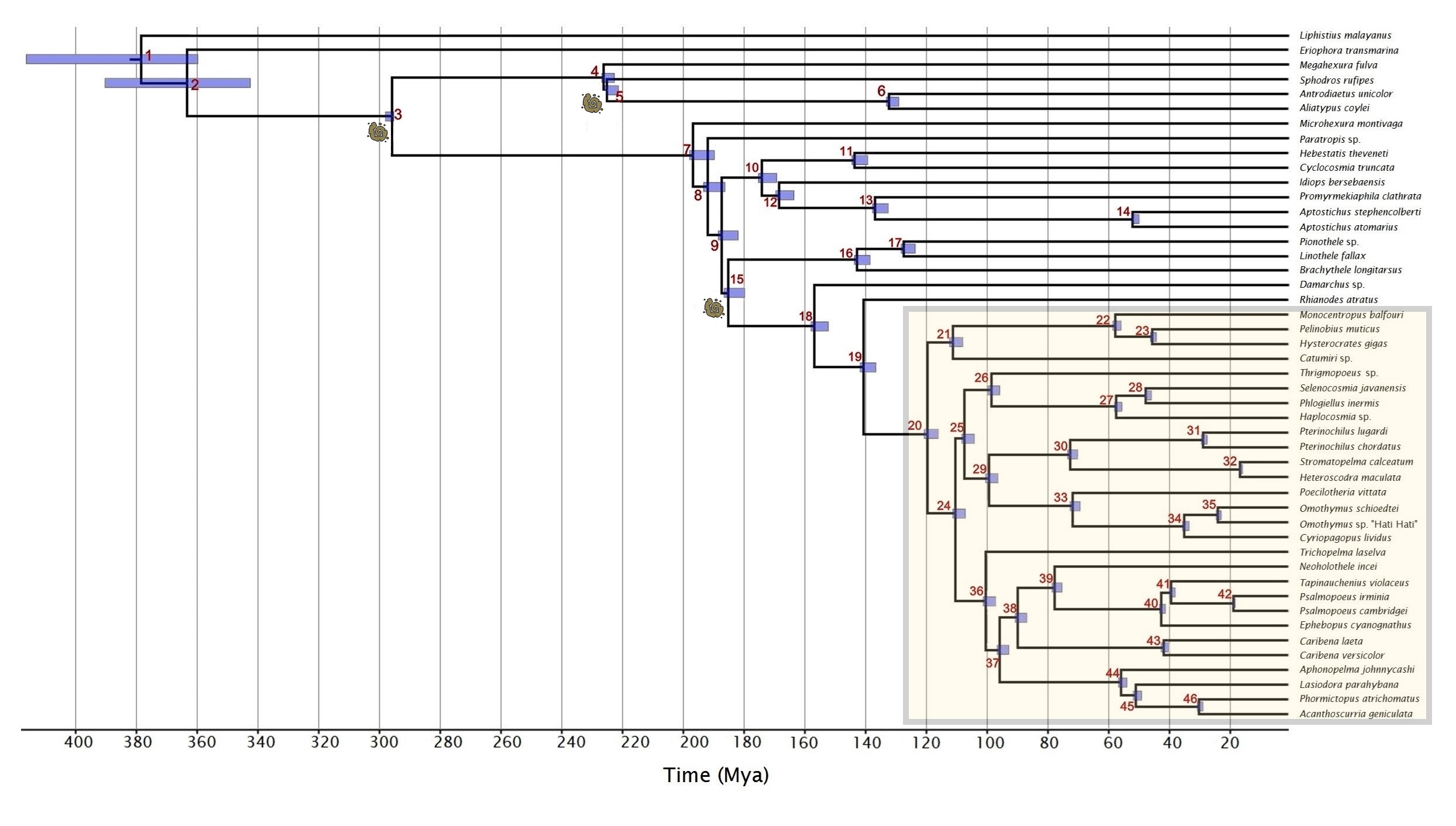

Supplement: Supplemental Information 9 — Error margins are shown as blue bars on all nodes, and a timescale is given in millions of years (Mya). Nodes are numbered, and fossil points are represented by small fossil icons. Theraphosidae are highlighted by the pallid yellow box. [file peerj-09-11162-s009.jpg]

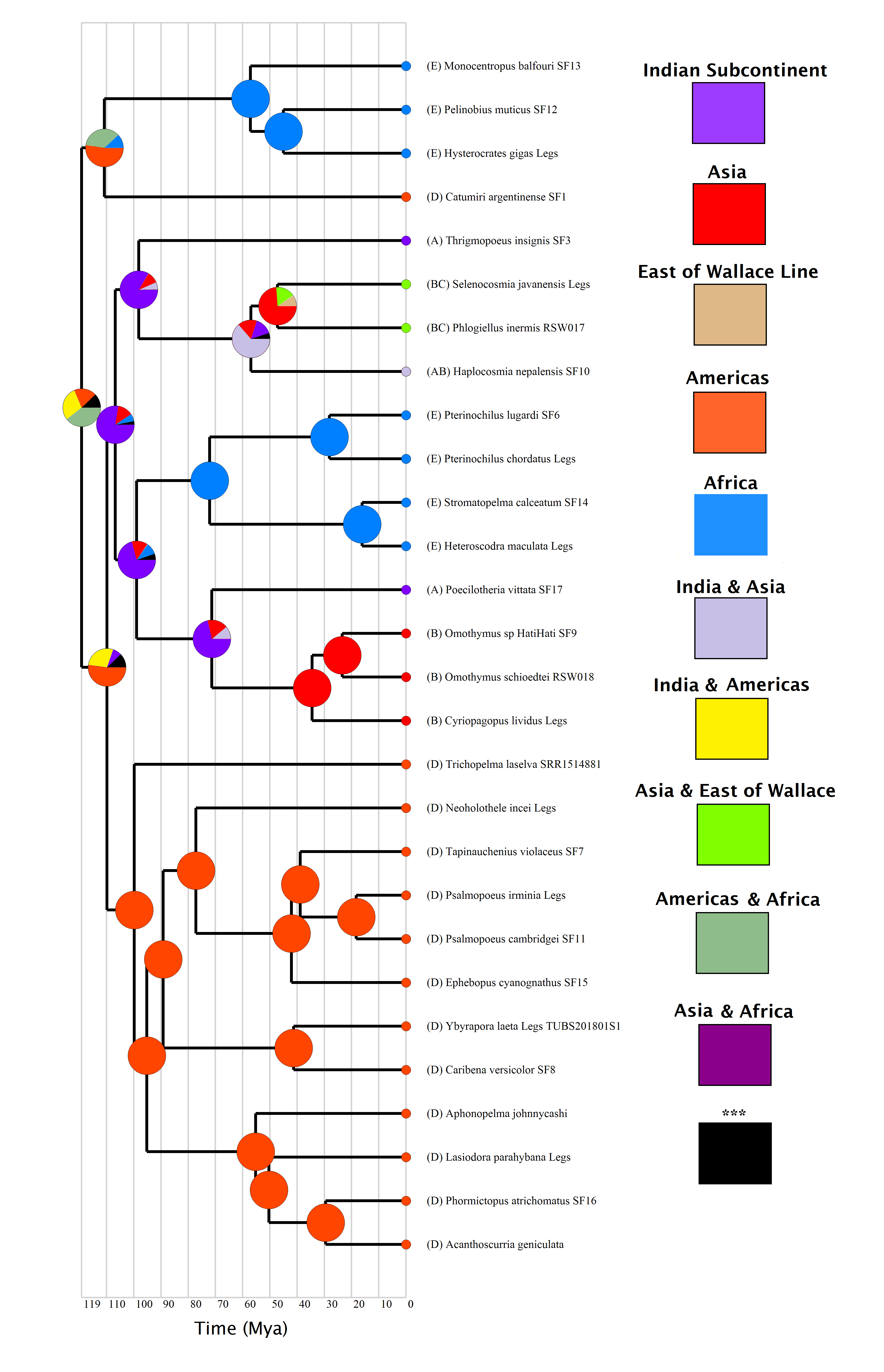

Supplement: Supplemental Information 10 [file peerj-09-11162-s010.png]

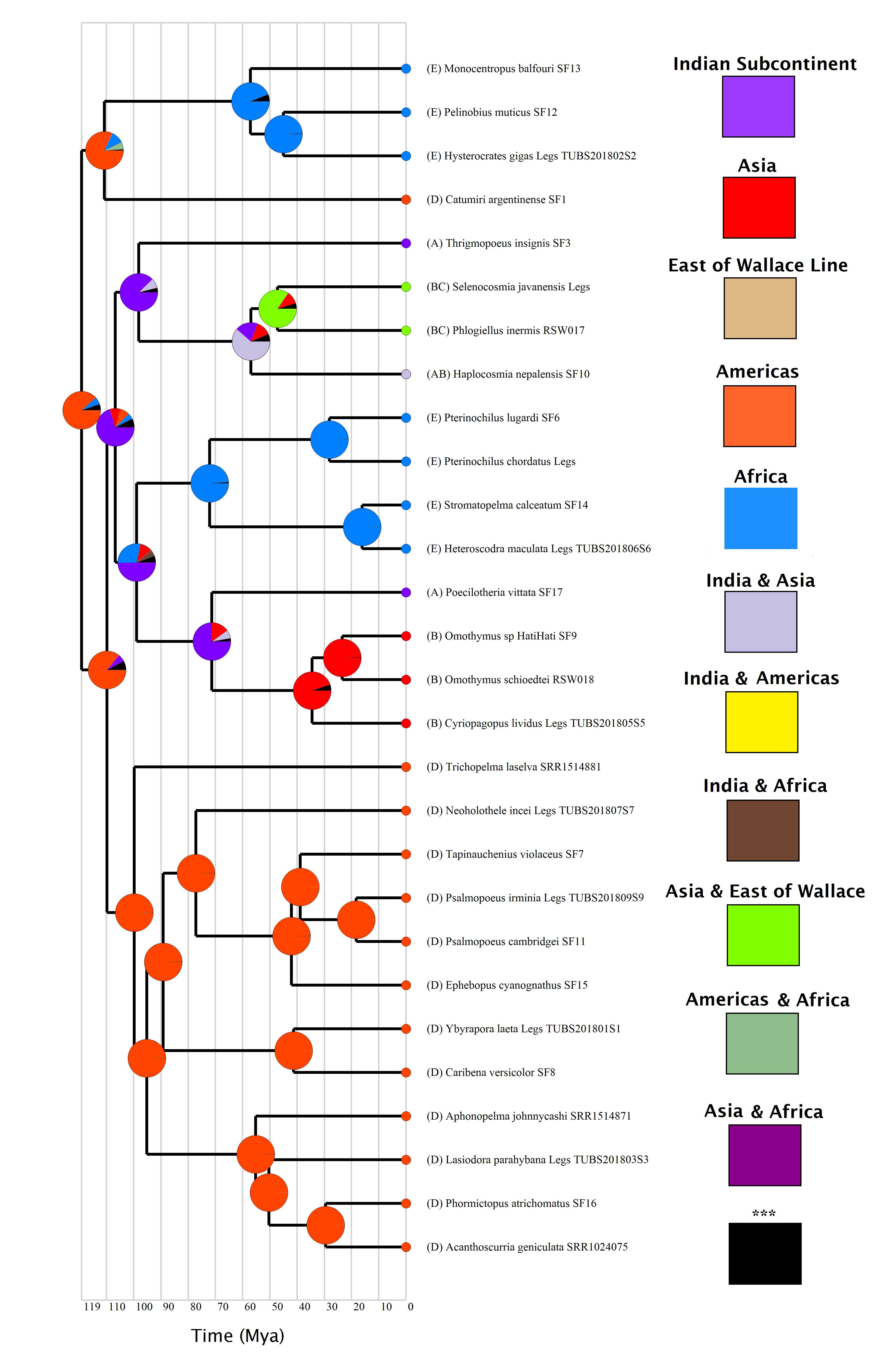

Supplement: Supplemental Information 11 [file peerj-09-11162-s011.png]
